# Assessment of influences of posterior rotation of the tibial condyles on the Insall-Salvati ratio

**DOI:** 10.1038/s41598-022-26459-6

**Published:** 2022-12-17

**Authors:** Ryuji Nagamine, Makoto Kawasaki, Kang-Il Kim, Akinori Sakai, Toru Suguro

**Affiliations:** 1 Nagamine Clinic for Rheumatology and Orthopaedic Surgery, Fukuoka, Japan; 2 Center of Artificial Joint and Rheumatism, Fukuoka Tokushukai Medical Center, Kasuga, Japan; 3 Department of Orthopaedic Surgery, School of Medicine, University of Occupational and Environmental Health, Kitakyushu, Japan; 4 Department of Orthopaedic Surgery, School of Medicine, Kyung Hee University, Seoul, Korea; 5 Japan Research Institute of Artificial Joint, Tokyo, Japan

**Keywords:** Anatomy, Musculoskeletal system

## Abstract

The positional relationship between patellar and femoral articular surfaces may vary according to the degree of posterior rotation of the tibial condyle, which may influence the patellar configuration. We hypothesized that the configuration of the patella has a rhomboid transformation similar to that of the tibial condyle. This cohort study included 313 patients with knee pain who underwent lateral-view knee digital radiography. The length of the long axis, short axis of the patella, and patellar tendon length of the patellofemoral joint were measured. The patella axis ratio (length of long/short axis) as patellar configuration and Insall-Salvati ratio were calculated. Correlations between the configuration of the tibial condyle and the three length parameters and the Insall-Salvati ratio were assessed. Posterior rotation and the rhomboid transformation of the tibial condyle were positively correlated with the length of the long axis of the patella and negatively correlated with the Insall-Salvati ratio. The more the tibial articular surface shifted posteriorly due to posterior rotation and rhomboid transformation of the tibial condyle, the longer the long axis of the patella was, and the smaller the Insall-Salvati ratio was. The long axis of the patella became longer due to rhomboid transformation, similar to the tibial condyle.

## Introduction

Until the end of bone growth, the tibial condyle changes its configuration. In the coronal plane, proximal tibiae have some degree of varus alignment (constitutional varus) that occurs at the physis^1^. Therefore, proximal tibia vara involves medial shift of the tibial articular surface^2^.

**Figure 1:**
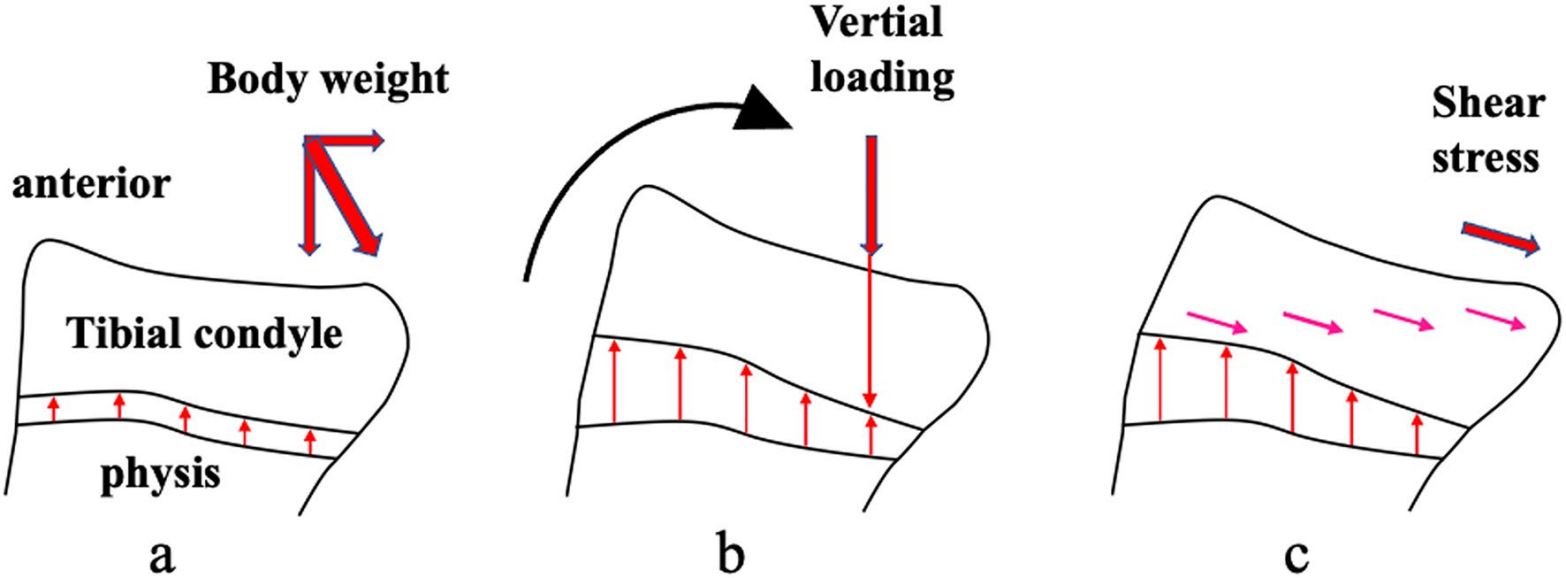
Schema of the tibial condyle with posterior rotation and rhomboid transformation during bone growth. (**a**) The tibial condyle moves superiorly with bone growth of the physis; however, body weight loading is applied. Loading has two directions (vertical loading and shear stress). (**b**) Vertical loading disturbs bone growth in the posterior area of the physis, and the tibial condyle rotates posteriorly with bone growth. (**c**) Shear strain by the shear stress induces rhomboid transformation of the tibial condyle.

In the sagittal plane, body weight loading has two directions (vertical loading and shear stress) (Fig. 1a). In the metaphysis, the bone grows in the anterior region and the growth is retarded in the posterior region by vertical loading based on the Hueter-Volkmann’s law (Fig. 1b); additionally, the tibial condyle rotates posteriorly. This phenomenon is defined as the constitutional posterior rotation^3^. The tibial condyle itself does not have a posterior slope of the tibial articular surface in more than 86% of knees^3^. The posterior slope is created due to the posterior rotation of the tibial condyle relative to the tibial shaft. In the tibial condyle, the shear strain due to shear stress changes the shape of the tibial condyle from a rectangle to a rhombus. This transformation was defined as the rhomboid transformation (Fig. 1c)^3^.

**Figure 2:**
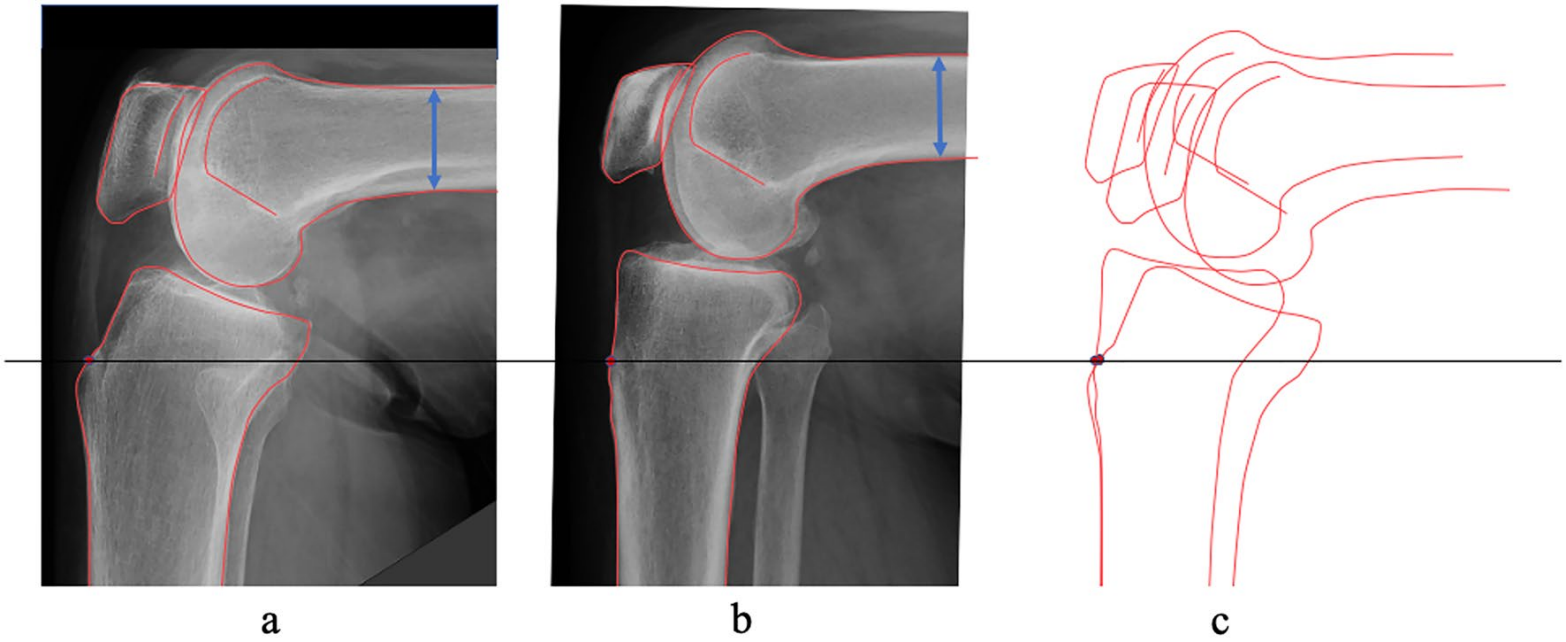
Influence of posterior rotation of the tibial condyle on the locations of the patella and the femoral condyle. (**a**) A tibia with a larger posterior slope, (**b**) A tibia with a minimum posterior slope of the tibial articular surface, (**c**) Contours of both tibiae were superimposed. With posterior rotation of the tibial condyle, the femoral condyle and patella shift posteriorly and inferiorly.

Posterior rotation and rhomboid transformation of the tibial condyle induce the posterior shift of the tibial articular surface (Figs. 1, 2)^3^. Though posterior rotation of the tibial condyle, the femoral condyle and patella shift posteriorly and inferiorly because the position of the femoral condyle is decided based on the tibial articular surface (Figs. 1, 2) (Supplementary Fig. 1)^4,5^. Congruency between the patellar and femoral articular surfaces may vary according to the degree of posterior rotation of the tibial condyle. Our hypothesis was the following: configuration of the patella might be constitutional, similar to the tibial condyle, and the patella might have rhomboid transformation due to shear stress of the patellofemoral joint. This study aimed to assess the influence of posterior rotation of the tibial condyle on the patellar configuration in the sagittal plane.

Clinically, the Insall-Salvati ratio is used frequently to assess the patella height in knees with patellofemoral joint disorders. The information presented in this study may contribute to the understanding of patellofemoral joint disorders.

## Methods

### Ethics statement and patient selection

The Institutional Review Board of Fukuoka Tokushukai Medical Center, in accordance with the Declaration of Helsinki 2013, approved the study protocol (Approval Number: 220103). Informed consent was obtained from all participants, and all experiments were performed according to relevant guidelines and regulations.

A retrospective review of all patients who visited our outpatient department due to knee pain between January 2017 and June 2020 was performed. In total 392 participants were included; cases with lateral osteoarthritis, patellofemoral arthritis, post-traumatic arthritis, or rheumatoid arthritis were not included in this study due to a possibility of having malrotation of the tibia relative to the femur. In addition, cases of bone defects or destruction of articular surfaces were not recruited. Consequently, 79 cases were excluded from this study, and 313 knees in 313 cases were assessed. This study included 245 women and 68 men. The mean age was 74.2 years old (range, 42–91 years). The mean height and weight were 153.5 cm and 60.2 kg respectively.

### Digital radiograph evaluation

**Figure 3:**
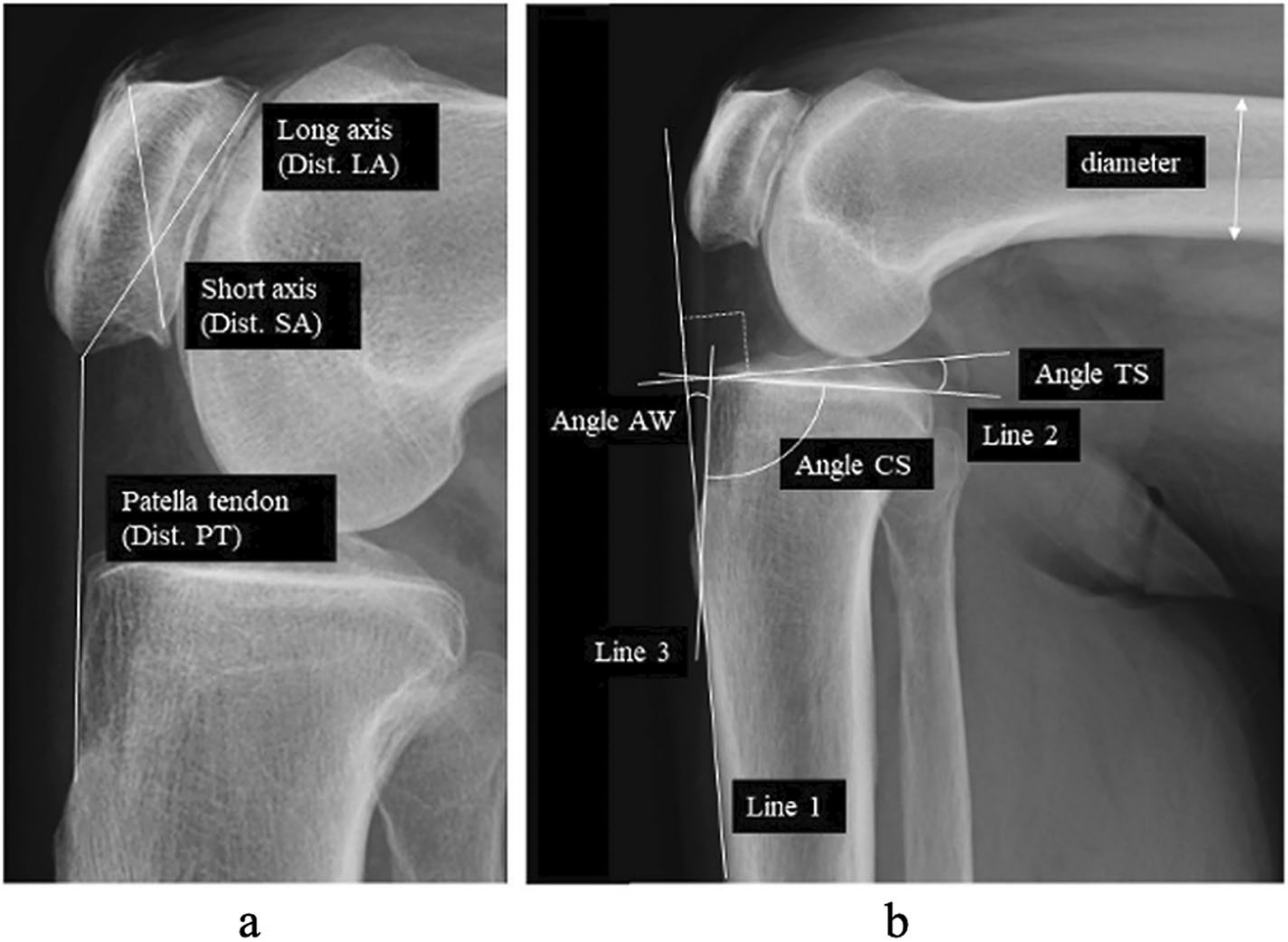
Parameters on lateral view radiograph of the knee. (**a**) Dist. LA: length of the long axis of the patella, Dist. SA: length of the short axis of the patella, Dist. PT: length of the patellar tendon, (**b**) Angle AW represents the angle of posterior rotation of the tibial condyle relative to the anterior cortex of the tibial shaft. Angle TS represents the posterior slope angle relative to the anterior cortex of the tibial shaft. Angle CS represents the degree of rhomboid transformation of the tibial condyle.

Lateral view digital radiographs of the knees were used for this study (Fig. 3). The flexion angle between the femur and the tibia was expressed using the anterior cortex of the shaft. The mean angle between the anterior cortex of the distal femoral shaft and the anterior cortex of the tibial shaft was 71.0 ± 20.7° (range 31.9–111.4°). Before the analysis of the relationship between tibial condyle parameters and patella parameters, the correlation between the flexion angle and all parameters was assessed. There was no correlation between flexion angle and any patella parameter including the Insall-Salvati ratio (Supplementary Fig. 2). The X-ray beam was centered on the knee. The X-ray tube was fixed at a source-to-image distance of 100 cm. An 8 × 10-inch cassette^2^ was placed immediately behind the participant, and a digital radiograph (Toshiba, Japan) was taken. A setting of 200 mA and a voltage of 50 kV was used. Regarding true lateral view, radiographs of the distance between the most posterior points of the lateral and medial femoral posterior condyles were 2 mm or less and used for the measurements (Fig. 3).

Digital radiographs were analyzed using computer software (ShadeQuest/ViewR, Yokokawa Medical Solutions Corporation, Japan). The long and the short axes were drawn on the patella and patellar tendon (Fig. 3a). The short axis was between the edge of the posterior and inferior corners and that of the anterior and superior corners. Patella tendon length (Dist. PT), length of the long axis of the patella (Dist. LA), and length of the short axis (Dist. SA) were measured. The value was expressed as a percentage of the diameter of the femur diaphysis. The patella axis ratio was calculated.

$${\text{Patella axis ratio}}\, = \,{\text{Dist}}.{\text{ LA}}\,/\,{\text{Dist}}.{\text{ SA}}.$$

**Figure 4:**
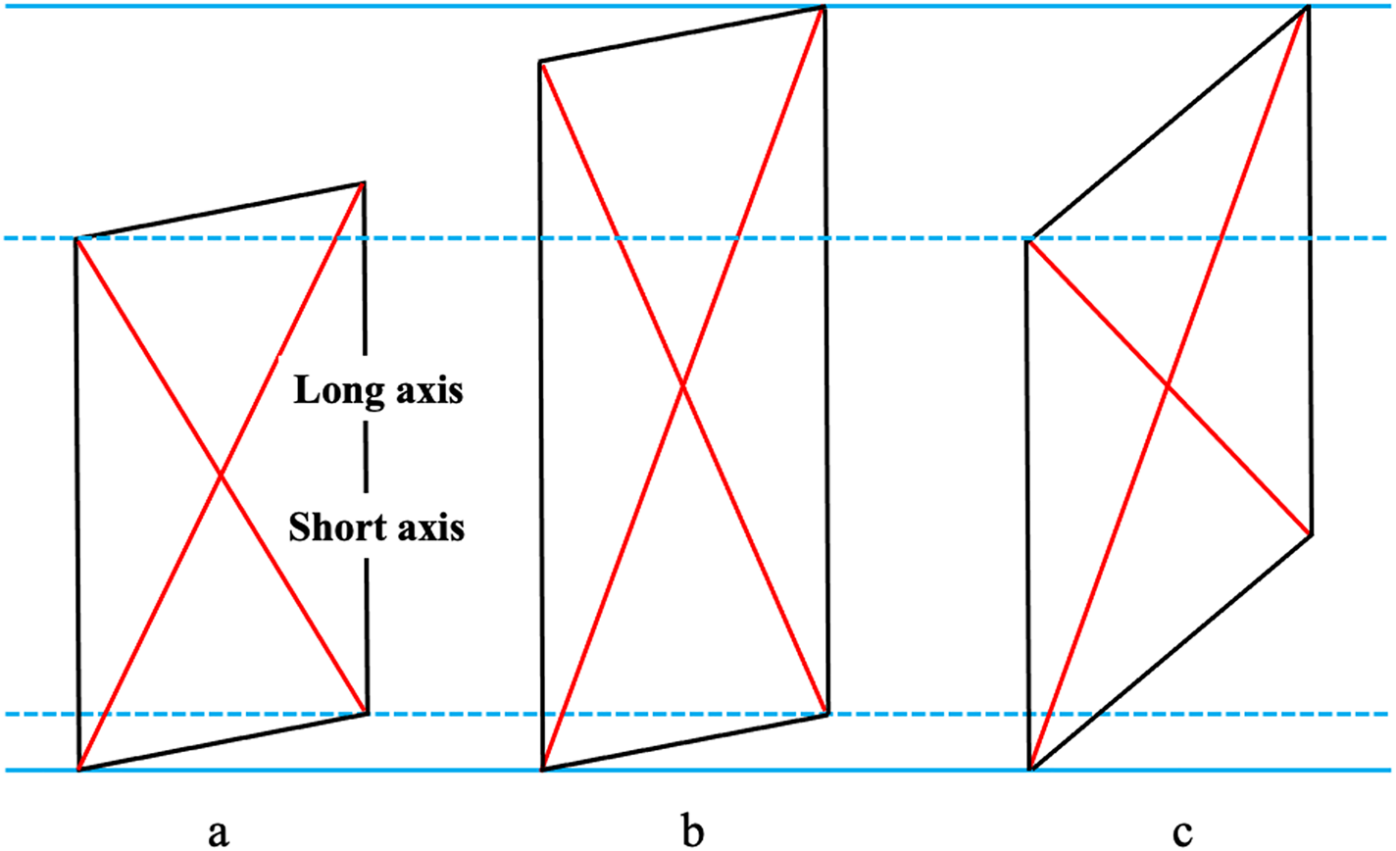
Schema of the long and short axes of the patella. (**a**) The long axis and the short axes are drawn, and the patella axis ratio is calculated. (**b**) With the simple elongation of the patella, both the long and short axes are getting longer and the patella axis ratio is getting smaller. (**c**) With rhomboid transformation of the patella, only the long axis is getting longer. The patella axis ratio is getting larger.

This ratio was defined for this study. The patella axis ratio was used to examine whether the patella had a rhomboid transformation. If the patella had a rhomboid transformation, Dist. LA was lengthened relative to that of Dist. SA, and Dist. LA had a positive correlation with the patella axis ratio (Fig. 4c). If the patella had a simple elongation (Fig. 4a,b), Dist. SA was closer to Dist. LA, and Dist. LA was negatively correlated with the patella axis ratio. If the configuration of the patella did not change, then Dist. LA was not correlated with the patella axis ratio.

The Insall-Salvati ratio, which is most commonly used to assess patellar height^6^, was also calculated. Reliability of Dist. SA was assessed using intraclass correlation coefficients. One of the authors measured Dist. SA three times and assessed the intra-observer reproducibility in 40 participants. Three of the authors measured Dist. SA independently and assessed the inter-observer reproducibility in 40 participants. The reproducibility method was blinded, and the readers performed all measurements without knowledge of prior results: all markings were erased for each reader. Intra-observer agreement of Dist. SA was 0.94 (P < 0.01). Inter-observer agreement of Dist. SA was 0.91 (P < 0.01).

Three angles were measured on the proximal tibia using the same method previously described (Fig. 3b)^3^. Three lines were drawn along the anterior cortex of the tibial shaft (Line 1), along the middle one-third of the medial articular surface (Line 2), and along the anterior wall of the tibial condyle (Line 3). Angle AW was the angle between Line 1 and Line 3. During bone growth, bone grows in the anterior part of the metaphysis and the growth is disturbed in the posterior part by the vertical loading of the body weight, and the tibial condyle rotates posteriorly (Fig. 1a,b). Therefore, Angle AW represented the posterior rotation angle of the tibial condyle relative to the anterior cortex of the tibial shaft. Angle TS was the angle between the line perpendicular to Line 1 and Line 2. Angle TS represented the posterior slope angle of the tibial articular surface relative to the anterior cortex of the tibial shaft. Angle CS was the angle between Line 2 and Line 3. The configuration of the tibial condyle changes from a rectangular to a rhombus due to the shear stress of body weight (Fig. 1c). Angle CS represented the degree of rhomboid transformation of the tibial condyle.

One of the authors measured all parameters three times, and the average of the three values was used for the analysis. Correlation among the parameters was assessed.

### Statistical analysis

Regression analysis was performed to assess the relationships between each parameter. We performed a power analysis and found that a minimum of 194 cases were required to perform simple correlation analyses (α = 0.05, power = 0.08, effect size = 0.4). All statistical analyses were performed using JMP version 14.2 (SAS Institute Inc., Cary, NC, USA). Statistical significance was set at P < 0.01.

## Results

The mean (± standard deviation [SD]) diameter of the femur diaphysis, Dist. LA, Dist. SA, and Dist. PT were 29.8 ± 2.4 mm, 141.9 ± 10.4, 100.6 ± 9.1 and 141.7 ± 15.2, respectively. The mean (± SD) patellar axis ratio and Insall-Salvati ratio were 1.4 ± 0.1 and 1.0 ± 0.1, respectively. The means (± SDs) of Angle TS, Angle AW, and Angle CS were 9.6 ± 3.7°, 15.5 ± 6.6° and 95.9 ± 5.5°, respectively.

**Figure 5:**
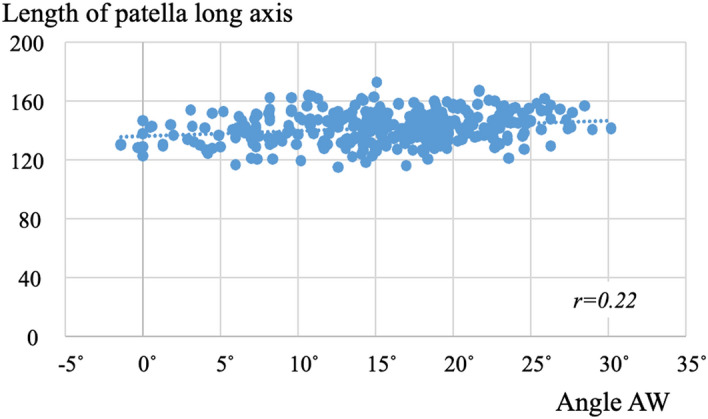
Correlation between Angle AW and length of the long axis of the patella. The more the tibial condyle rotates posteriorly, the longer the long axis of the patella becomes.

**Figure 6:**
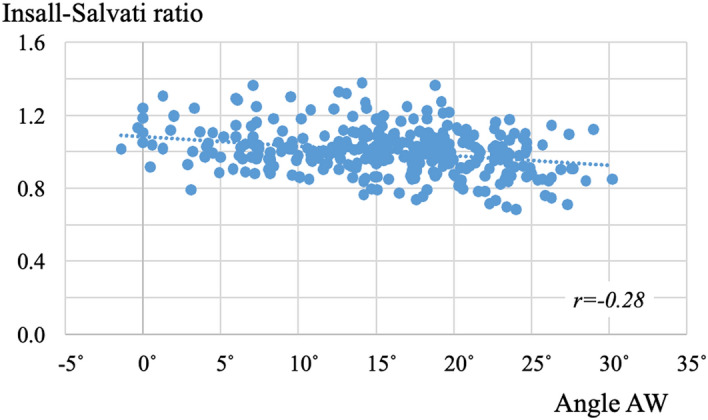
Correlation between Angle AW and the Insall-Salvati ratio. The more the tibial condyle rotates posteriorly, the smaller the Insall-Salvati ratio becomes.

Dist. LA was positively correlated with Angle AW and Angle CS (r = 0.22 and 0.21, respectively) (Fig. 5). Dist. PT was not correlated with Angle AW or Angle CS (r = − 0.17 and − 0.14, respectively). The Insall-Salvati ratio was negatively correlated with Angle AW and Angle CS (r = − 0.28 and − 0.24, respectively) (Fig. 6). The more tibial articular surface shifted posteriorly due to posterior rotation and rhomboid transformation of the tibial condyle, the longer the long axis of the patella was, and the smaller the Insall-Salvati ratio was.

Dist. LA was positively correlated with the patella axis ratio (r = 0.33). The longer the Dist. LA was, the higher the patella axis ratio was. The patella with longer a longer axis tended to have a rhomboid configuration.

Angle AW was positively correlated with Angle CS and Angle TS (r = 0.83 and 0.55, respectively). Angle CS was not correlated with Angle TS (r = − 0.02). No correlation was found between age, height, or weight, and Dist. LA, Dist. SA, Dist. PT, Angle AW, Angle CS, and Angle TS.

## Discussion

This was the first study to focus on the relationship between the configuration of the tibial condyle and the patella. It has been proven that the posterior rotation and rhomboid transformation of the tibial condyle shift the tibial articular surface, patella, and femoral condyle posteriorly and distally^5^. In that study, the radiograph was taken with the knee flexed at 90° to assess the position of the patella and femoral condyle relative to the tibial tubercle in all knees. The contact area and pressure in the patella-femoral joint may be different in individuals according to the degree of tibial condyle posterior rotation. Figure 2 shows differences in the locations of the patella and femoral condyle between knees with larger posterior rotation of the tibial condyle and those without posterior rotation when the anterior cortex of the tibial shaft and patellar tendon attachment were matched. The mean (± SD) the Insall-Salvati ratio was 1.0 ± 0.1 regardless of knee flexion angle. Larger Angle AW and Angle CS influenced the length of the long axis of the patella. An elongated patella with a superior shift of the patellar articular surface may maintain better congruency between the femoral articular surfaces that moved posteriorly and inferiorly. Elongation of the patella may be constitutional because no correlation was found between Dist. LA and age, height, or weight. Dist. LA was positively correlated with the patella axis ratio, which proved that the patella with elongation of the long axis had a rhomboid transformation. Based on understanding the mechanics of the elasticity, it is possible to explain that shear stress by body weight loading to the patellar articular surface induces rhomboid transformation of the patella, similar to the tibial condyle (Fig. 4)^3^. Further studies are necessary to assess the difference of the contact stress in the patella-femoral joint by the tibia condyle posterior rotation.

This study had several limitations. First, the knee joints were evaluated in patients with knee pain and not in healthy individuals. Therefore, the normal configuration of the tibial condyle and patella remains unclear. However, this study is important as it proved that the configuration of the tibial condyle was correlated with the configuration of the patella. Second, the short axis of the patella established in this study is not popular. The configuration of the anterior cortex of the patella is different among individuals, and it is difficult to set a reliable landmark on the anterior cortex of the patella. The influence of the configuration of the distal part of the patella on the Insall-Salvati ratio has also been reported^7^. Therefore, it is difficult to assess the location of the patellar articular surface relative to the anterior surface of the patella. Although the patella axis ratio cannot assess the location of the articular surface relative to the anterior cortex, this ratio can show the configuration of the patella. If the patella was rectangular, the patella axis ratio was 1.0. With the patella changing its configuration from rectangular to the rhomboid, the patella axis ratio increases. The results of this study showed that Dist. LA was positively correlated with the patella axis ratio, and it can be concluded that the patella with the elongation of the long axis had a rhomboid transformation. Third, if the patella had malrotation in three-dimensional coordinate systems, the values of patella parameters may be different. In this study, patients with lateral osteoarthritis, patellofemoral arthritis and post-traumatic arthritis were not included because the position and the rotation angles of the patella may be different; additionally, true lateral view radiographs were taken. The distances between the most posterior points of the lateral and medial femoral posterior condyles were 2 mm or less in all radiographs. With these conditions, the position and rotation angle of the patella were thought to be constant.

The results of this study may be clinically relevant such as for the evaluation of the patella height and the evaluation of clinical results of total knee arthroplasty (TKA). The normal range between 0.8 and 1.2 to examine patella alta or baja expressed by the Insall-Salvati ratio may not be suitable for all cases. It has been reported that the range of the Insall-Salvati ratio is different among races^8–10^. Apostolopoulos et al. reported that the Insall-Salvati ratio was 0.86 in groups whose daily activities included squatting of the traditional Muslim praying position and was 1.1 in groups who were non-Muslim^9^. It has been reported that the traditional Asian way of living such as sitting straight (Japanese seiza) may impair bone growth at the proximal metaphysis in childhood in the coronal plane, and may induce proximal tibia vara^11^. Squatting of the Muslim praying position may influence of the configuration of the tibial condyle in the sagittal plane. Lin et al. reported that the Insall-Salvati ratio was significantly smaller in a group with anterior cruciate ligament (ACL) injury compared with a control group with the internal disorder of the knee^12^. It is commonly recognized that a large posterior tibial slope can be a risk factor for ACL injury^13–15^. The results of small values of the Insall-Salvati ratio reported in previous studies^8,12^ may not have meant patella baja relative to the femoral condyle. To assess patellar height using the Insall-Salvati ratio, the configuration of the proximal tibia and the location of the patellar articular surface and the femoral condyle should also be assessed. Differences in the location of the three bones may indicate some degree of kinematic problem of the knee joint with larger posterior rotation of the tibial condyle, which may be a risk factor for ACL injury.

Differences in the location of the three bones also influence the sagittal alignment of the patella and the femoral components, and tibial component in TKA. A certain number of patients complain of discomfort in implanted knees^16–18^. Further studies to evaluate the kinematics of implanted knees taking the degree of the tibial condyle posterior rotation, true patella height, and the difference in the location of the three bones, may be necessary to assess the dissatisfaction of the patients after TKA.

## Conclusions

The long axis of the patella became longer and the Insall-Salvati ratio became smaller due to the posterior rotation and rhomboid transformation of the tibial condyle. The long axis of the patella became longer due to rhomboid transformation, similar to the tibial condyle. Posterior rotation and rhomboid transformation of the tibial condyle might influence the length of the long axis of the patella and the Insall-Salvati ratio.

## Supplementary Information

Supplementary Figure 1.

Supplementary Figure 2.

## Data Availability

The dataset generated during the current study are not publicly available but are available from the corresponding author on reasonable request.
