# Supplementary figures and images for: Assessment of influences of posterior rotation of the tibial condyles on the Insall-Salvati ratio
Source: Sci Rep. 2022 Dec 17;12:21850. doi: 10.1038/s41598-022-26459-6 (PMC9759518; doi:10.1038/s41598-022-26459-6)

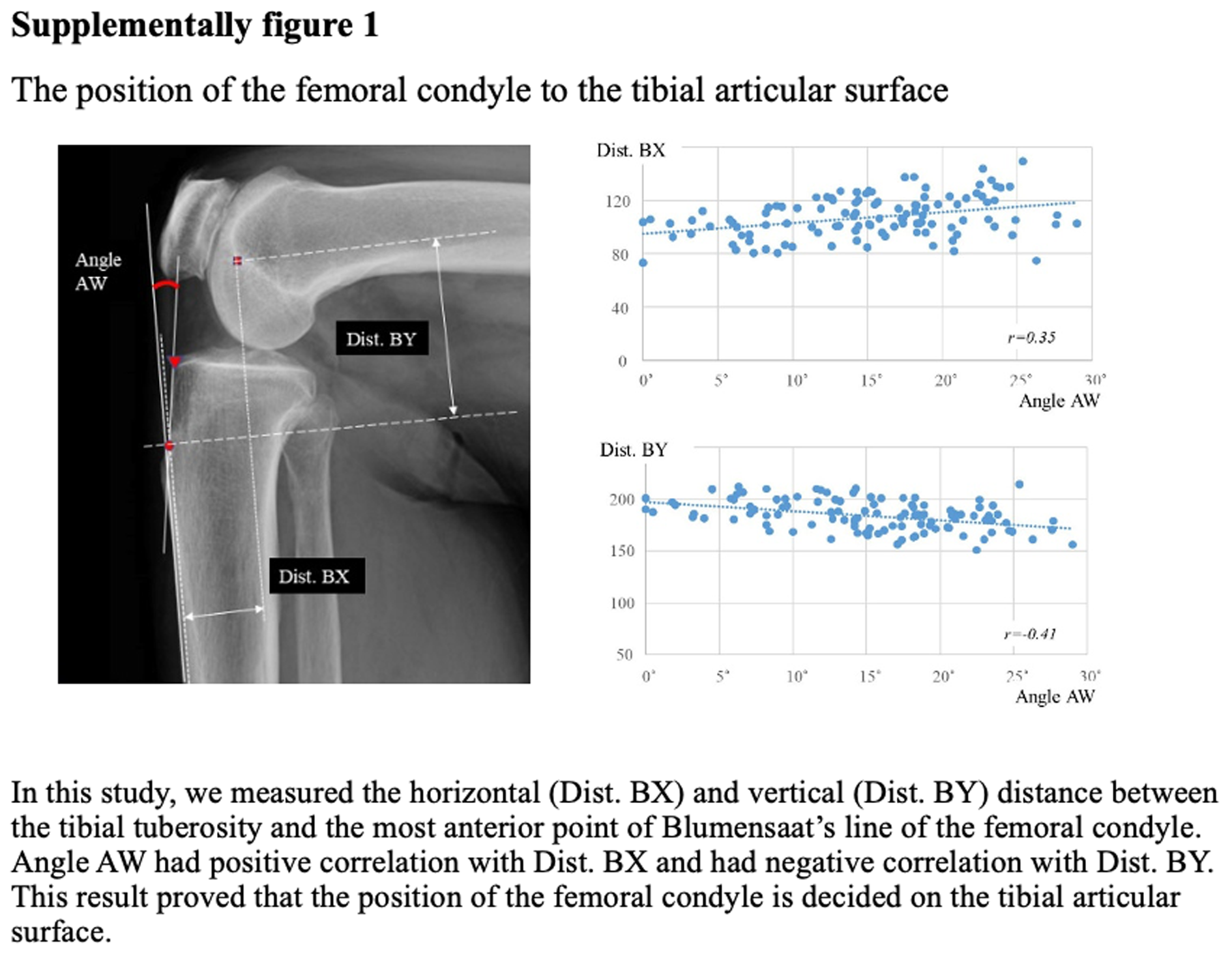

Supplement: Supplementary file 1 — Supplementary Figure 1. [file 41598_2022_26459_MOESM1_ESM.tif]

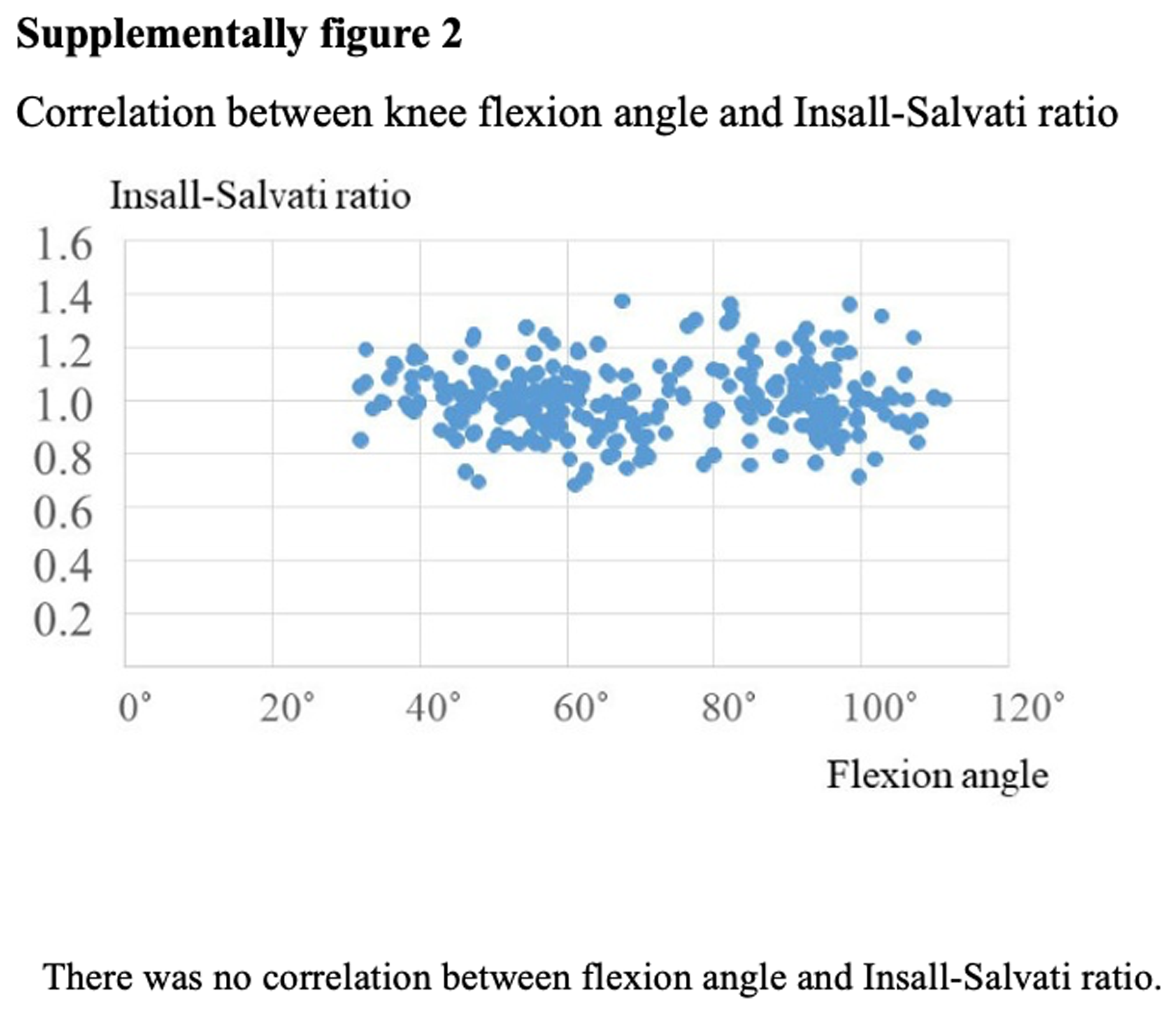

Supplement: Supplementary file 2 — Supplementary Figure 2. [file 41598_2022_26459_MOESM2_ESM.tif]
